# A fluorescent photoimmunoconjugate for imaging of cholesteatoma

**DOI:** 10.1038/s41598-022-22072-9

**Published:** 2022-11-19

**Authors:** Samuel Early, M. Ahsan Saad, Srivalleesha Mallidi, Amer Mansour, Richard Seist, Tayyaba Hasan, Konstantina M. Stankovic

**Affiliations:** 1 Department of Otolaryngology – Head and Neck Surgery and Eaton-Peabody Laboratories, Massachusetts Eye and Ear, Boston, MA USA; 2 Department of Otolaryngology – Head and Neck Surgery, Harvard Medical School, Boston, MA USA; 3 Department of Otolaryngology – Head and Neck Surgery, University of California San Diego Medical Center, San Diego, CA USA; 4 Wellman Center for Photomedicine, Massachusetts General Hospital, Boston, MA USA; 5 Department of Biomedical Engineering, Tufts University, Medford, MA USA; 6 Department of Otolaryngology – Head and Neck Surgery, Stanford University School of Medicine, Stanford, CA USA

**Keywords:** Preclinical research, Diagnostics, Fluorescence imaging, Skin diseases

## Abstract

Cholesteatoma is a potentially serious complication of chronic ear infections and requires surgical intervention for definitive management. Long-term complications include a frequent need for repeat surgical intervention for disease recurrence, and techniques to improve efficacy of single-stage surgery are an important area of continued research. This study investigates a novel application of the photosensitizer immune conjugate (PIC) cetuximab-benzoporphyrin derivative (Cet-BPD) for in vitro localization of human cholesteatoma tissue, coupled with an in vivo safety study for middle ear application of Cet-BPD in a murine model. In fresh human cholesteatoma tissues, Cet-BPD demonstrates selective localization to the hyperplastic squamous cell tissue associated with cholesteatoma, without localizing to other tissues such as middle ear mucosa. Applied to the murine middle ear, Cet-BPD does not demonstrate any deleterious effect on murine hearing when assessed by any of auditory brainstem response (ABR) thresholds, distortion product otoacoustic emission thresholds, or ABR wave I amplitudes. These findings demonstrate the technical promise and encouraging safety profile for the use of PICs for intraoperative localization and treatment of cholesteatoma.

## Introduction

**Figure 1:**
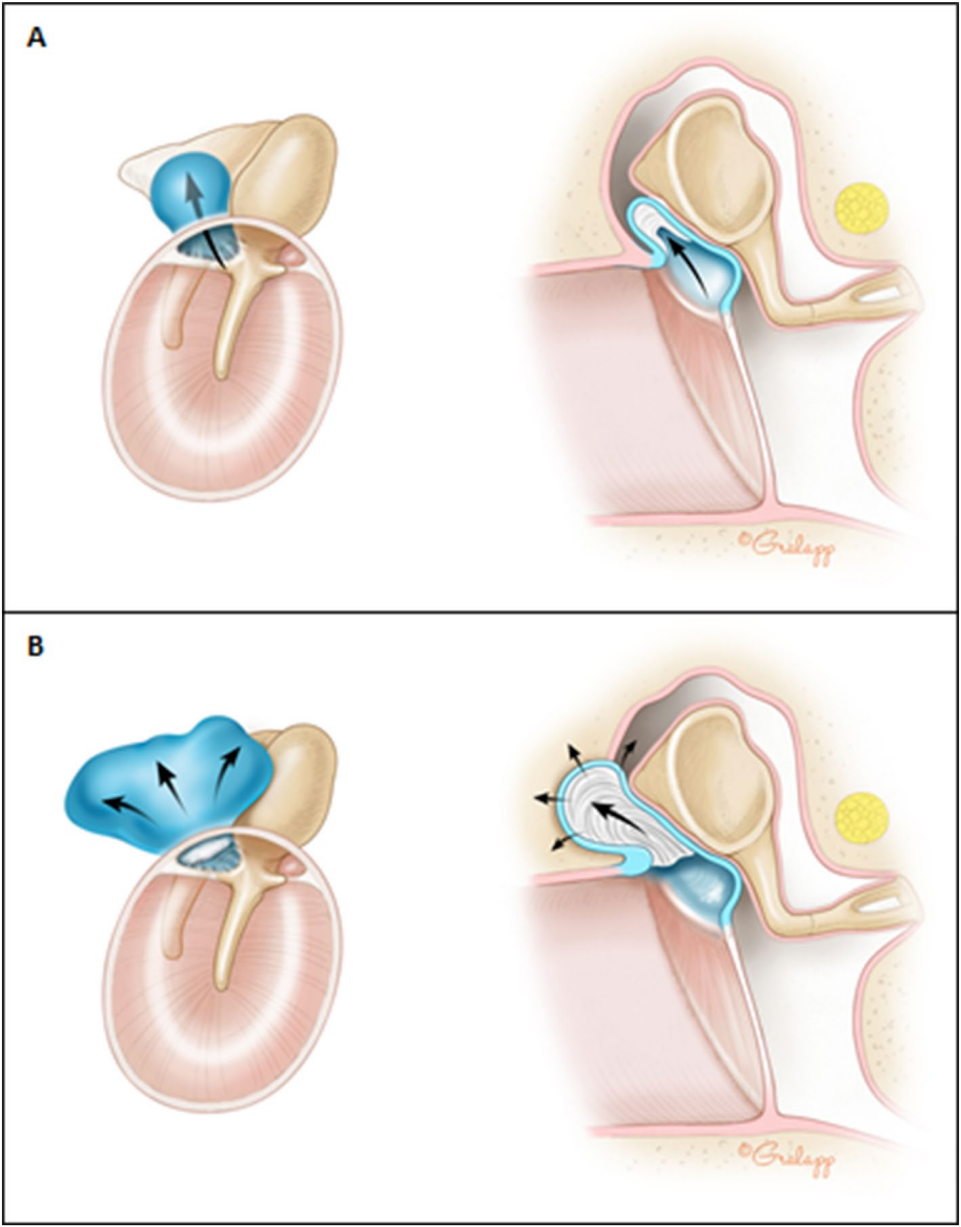
Graphic representation of typical cholesteatoma progression in the middle ear space, beginning with (**A**) attic retraction pocket of the pars flaccida portion of the tympanic membrane followed by (**B**) gradual progression into the middle ear and mastoid space with erosion of ossicles and potentially other bony structures as well. Of note, cholesteatoma can also commonly be acquired from a tympanic membrane perforation, or may also be congenital in origin. Illustration Chris Gralapp^3^.

Cholesteatoma is a common complication of chronic ear infections and typically requires repeat surgical intervention to eradicate entirely. Infection of the middle ear, otitis media, is the most common cause for visits to the pediatrician, with 80% of children worldwide developing an episode of acute otitis media before the age of 3, and 40% of children having six or more recurrences by the age of 7^1^. Otitis media costs the US healthcare system alone an estimated $2.88 billion annually, not accounting for indirect costs such as missed time from work and school^2^. While antibiotics are often effective in the acute setting, they have limited ability to prevent recurrent infections. Inflammatory changes associated with chronic ear infections promote growth of cholesteatoma, which is a skin cyst that develops from the surface of the tympanic membrane and grows into the middle ear (Fig. 1)^3^. This cyst can slowly accumulate excessive keratin debris, progressively eroding into the bony structures of the middle ear and the normally aerated spaces of the mastoid bone^4^. Less commonly, cholesteatoma can be acquired secondary to tympanic membrane perforation, or can be congenital, however the composition of abnormal tissue is the same^5,6^. In regions of the world where poor hygiene, malnutrition, poverty, and smoking are prevalent and access to healthcare is limited, rates of cholesteatoma can be as high as 1.5% of all children, with associated high risk for complications including hearing loss, facial paralysis and meningitis^7–10^.

Surgery remains the only definitive cure for cholesteatoma, making cholesteatoma removal a common reason for ear surgery. Even for a skilled surgeon, however, it can be difficult to remove the disease entirely at the time of initial surgery due to tight adherence of the cholesteatoma matrix to critical structures of the middle ear, such as the facial nerve, the ossicles or dura of the temporal lobe^4,11^. Rates of cholesteatoma recurrence after surgery have been reported as high as 30%, and 11% of patients will require multiple revision surgeries to eradicate the disease^12–16^. The need for multiple surgeries is of particular concern in the pediatric population, where the relationship between repeat anesthesia episodes and possible negative effects on brain development has come under greater scrutiny in recent years^17,18^. Intraoperative application of sodium 2-mercaptoethanesulfonate has been shown to be effective at reducing incidence of disease recurrence, and new techniques such as endoscopic middle ear surgery have been shown to reduce the trauma associated with surgical excision of cholesteatoma, as well as the need for repeat surgery^14–16,19^. Even the most aggressive surgical strategy, however, a canal wall down mastoidectomy, retains a recurrence rate of between 2–5% while at best committing the patient to a small, permanent conductive hearing loss and a lifetime of scheduled ear cleanings^16^. In less-developed regions of the world, the priority of removing disease often takes precedence over saving hearing—surgeons must be very aggressive with the primary surgery because patients are more likely to present with complications and are less likely to be able to follow up in clinic, making a staged surgery to restore hearing not always feasible^20^.

Techniques to better localize cholesteatoma intraoperatively during primary surgeries have the potential to eliminate need for revision surgeries, and to increase surgeon confidence to perform primary ossicular chain reconstruction in some cases. For patients in whom follow up may be inconsistent, this improved confidence could obviate the need to prioritize ear safety over restoration of hearing.

A promising candidate marker for intraoperative localization of cholesteatoma is EGFR, since skin cysts are known to overexpress this marker compared to adjacent tissues of the head and neck^11,21^. Fluorescent epidermal growth factor receptor (fEGFR) antibodies are drugs that localize selectively to tissues with squamous hyperplastic processes with overexpression of EGFR, and fEGFR antibodies have been used in head and neck oncology to improve localization accuracy and effectiveness of surgical excision^22–25^. Further innovations with other photosensitizer immune conjugates (PICs), most notably cetuximab-benzoporphyrin derivative (Cet-BPD), have further refined ability for localization of EGFR-positive tumors cells through selective activation of fluorescence following metabolism and cleavage of the photosensitizer, with secondary advantage of targeted therapeutic tumor ablation through photoactivation of the immune conjugates once they are appropriately localized^26–29^.

All of these innovative markers from the world of oncology imaging and phototherapy have potential for application to intraoperative imaging and localization of cholesteatoma as well. In this paper, we demonstrate that PICs can accurately localize the squamous hyperplasia of cholesteatoma in vitro, and that these conjugates are not ototoxic in vivo.

## Methods

### Immune conjugate preparation

Conjugates of BPD and Cetuximab monoclonal antibody were prepared in 40% DMSO in accordance with prior protocol^30–32^. Briefly, the N-hydroxysuccinimide ester of BPD was reacted with antibody, in a 9:1 (dye to antibody) molar ratio, which had previously been polyethylene glycolated. The resulting immunoconjugate was purified using a Zeba spin desalting column (Thermo Fisher Scientific, Waltham, MA, United States) and the final DMSO content was reduced to 5% by subjecting it to buffer exchange using a 30 kDa MWCO Amicon Ultra centrifugal filter unit (Millipore Sigma, Burlington, MA, United States). Purity of immunoconjugates was assessed by gel fluorescence imaging analysis following SDS polyacrylamide gel electrophoresis, with less than 5% residual unconjugated BPD impurity^33^. The molar ratio of BPD to antibody was measured using the Pierce BCA Protein Assay (Thermo Fisher Scientific) and BPD absorbance spectroscopy at 690 nm. Maximum antibody conjugate concentration achievable by this method was 3.8 mg/ml in 5% DMSO. Cetuximab conjugated to Alexa-647 fluorescent dye (Cet-AF647) was prepared in a similar manner using a 4:1 (dye to antibody) ratio in phosphate-buffered saline.

### Ototoxicity evaluation in murine model

Ototoxicity tests were performed in adult CBA/CaJ mice at 1 mg/ml concentration for the PIC, diluted from stock solution in phosphate-buffered saline. Twelve mice were anesthetized with a mix of xylocaine and ketamine and the left bulla accessed by retroauricular approach to visualize the round window. In six experimental mice, a 5 μl volume of PIC solution was used to fill the middle ear space and confirmed to cover the round window niche, after which the bulla defect was repaired with rotational muscle graft and the postauricular incision closed with 7-0 Nylon sutures. In six control mice, the same approach and closure techniques were used, however with 5 µl phosphate-buffered saline applied to the middle ear space instead.

Auditory brainstem responses (ABRs) and distortion product otoacoustic emissions (DPOAEs) were recorded two weeks after surgery, as detailed previously^34,35^. Briefly, mice were anesthetized with intraperitoneal ketamine (100 mg/kg) and xylazine (10 mg/kg). Two miniature earphones serving as sound sources (CDMG15008-03A, CUI, Tualatin, OR, United States) and a microphone (FG-23329-P07, Knowles, Itasca, IL, United States) coupled to a probe tube within a custom acoustic system were used to measure sound pressure near the tympanic membrane. DPOAEs were measured as ear canal pressure in response to two tones presented into the ear canal (f1 and f2, with f2/f1 = 1.2 and f1 being 10 dB above f2) at half octave steps from f2 = 5.66 to 45.25 kHz, and in 5 dB intensity increments from 10 to 80 dB sound pressure level (SPL). DPOAE thresholds were defined as the f2 intensity required to generate a DP response 10 dB SPL over noise floor. Five millisecond tone pips were used to elicit ABR, which was measured between subdermal electrodes (adjacent to the ipsilateral incision, at the vertex, and near the tail), amplified 10,000 times and filtered (0.3–3.0 kHz). A total of 512 responses were recorded for each frequency and sound level and averaged using custom LabVIEW data-acquisition software run on a PXI chassis (National Instruments Corp., Austin, TX, United States). ABR waveforms stacked from lowest to highest SPL were assessed by intrinsic comparison matching with established murine ABR and DPOAE datasets and confirmed by visual inspection as the first threshold level at which a repeatable waveform was visually detected^36^. Institutional Review Board (IRB) approval was obtained from the Animal Care Committee at Massachusetts Eye and Ear and Massachusetts General Hospital (IRB 17-009). All animal experiments were performed in accordance with relevant guidelines and regulations, and are reported in accordance with the ARRIVE guidelines. All data analyzed during this study are included in this article and its supplementary information files.

### Tissue culture with PICs

Human tissue samples were collected from the operating room, either from patients undergoing cholesteatoma resection surgery or with skin available from benign surgical procedures for other otolaryngology indications. All tissues were collected immediately from the operating room and stored < 4 h in sterile saline before being placed in tissue culture. Tissue segments, whether cholesteatoma or normal skin, were microdissected to a target thickness of 1 mm so as to easily lie flat under the microscope for later analysis. Institutional Review Board (IRB) approval was obtained from the Human Studies Committee at Massachusetts Eye and Ear and Massachusetts General Hospital (IRB 19-017H), and written informed consent was obtained from all study subjects. All human tissue experiments were performed in accordance with relevant guidelines and regulations.

To create the experimental culture media, the immune conjugate stock solution was diluted to 100 μg/ml concentration in Dulbecco's Modified Eagle Medium/Nutrient Mixture F-12 (DMEM/F-12) culture media with 10% fetal bovine serum and 1% penicillin. The control solution for tissue incubation was simply DMEM/F-12 with 10% fetal bovine serum and 1% penicillin. All incubations were performed under minimal light-exposure conditions, with particular emphasis on avoiding *uv* light exposure given its potential to prematurely activate BPD. Tissues were cultured at 37 °C for 24 h with 5% ambient CO_2_, then rinsed in phosphate-buffered saline and fixed in 4% paraformaldehyde for imaging.

### Imaging of cholesteatoma tissues

Tissues incubated with PICs were mounted and imaged directly. Control tissues were divided into two portions, one imaged without any further processing to serve as a negative control for fluorescent labeling localization to EGFR-expressing tissues, and the second after staining with Cet-AF647 conjugate, to serve as a positive control for fluorescent labeling localization to EGFR-expressing tissues. Imaging was performed using a Leica TCS SP5 confocal microscope, with 10 μm cuts through tissues merged in a z-stack to obtain a z-axis projection image in the Leica software. Confirmatory histopathology was then performed to validate differential localization of PICs by tissue type.

### Statistical analysis

Statistical analysis was performed using GraphPad Prism 9.0.0 (GraphPad Software, Inc., San Diego, CA, United States) and Excel (Microsoft Corporation, Redmond, WA, United States). Comparison of hearing outcomes between study groups was assessed by unpaired *t-test*, with a probability value of p < 0.05 considered statistically significant.

## Results

### Safety tests in mice

**Figure 2:**
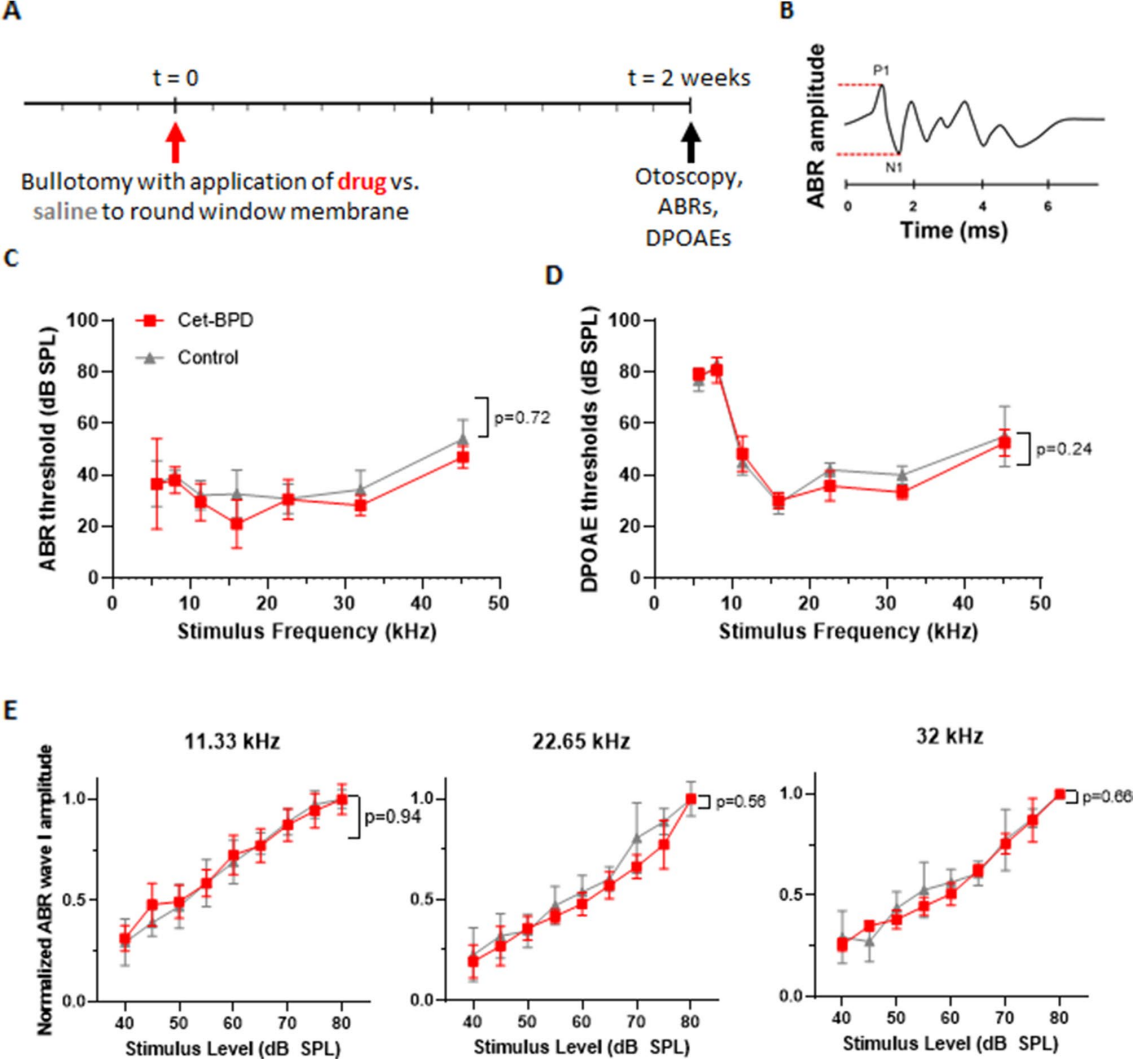
Cet-BPD PIC is not ototoxic. (**A**) Schematic timeline of the experimental protocol. (**B**) Schematic of ABR waveform; ABR wave I amplitude is measured between P1 and N1. (**C**) ABR thresholds and (**D**) DPOAE thresholds are plotted as a function of frequency for Cet-BPD group (red, N = 6 mice) and control group that received phosphate-buffered saline (gray; N = 5 mice). Data are represented as means + SEM. (**E**) ABR wave I amplitude, normalized to the amplitude of ABR wave I at 80 dB in the same animal, is plotted as a function of stimulus level for three representative frequencies for Cet-BPD group (red, N = 6 mice) and control group that received phosphate-buffered saline (gray; N = 5 mice). Data are represented as means + SEM.

Functional assessment of hearing, based on ABR and DPOAE testing two weeks after surgery, revealed no significant difference in hearing between operated vs. non-operated ears (N = 11 mice), and no significant difference in hearing between operated ears in mice treated with PIC solution (N = 6) vs. control solution (N = 5) (Fig. 2). ABR consists of several peaks and represents the summed electrical signal from cochlear neurons and higher order neurons in the brainstem. While ABR threshold testing is an excellent screening tool, wave I amplitude—calculated between the positive deflection (P1) and negative deflection (N1) (Fig. 2B)—is the most sensitive metric of cochlear nerve integrity. DPOAEs reflect function of outer hair cells. Cet-BPD treatment did not cause any significant change in ABR thresholds (Fig. 2C), DPOAE thresholds (Fig. 2D) or ABR wave I amplitude; the latter is shown for representative frequencies at which murine hearing is most sensitive (Fig. 2E). Of note, one mouse in the control group was excluded from the analysis because otomicroscopic examination two weeks post-surgery revealed a red, bulging tympanic membrane ipsilateral to the surgery, consistent in appearance with otitis media.

### Fluorescent imaging in cholesteatoma tissues

**Figure 3:**
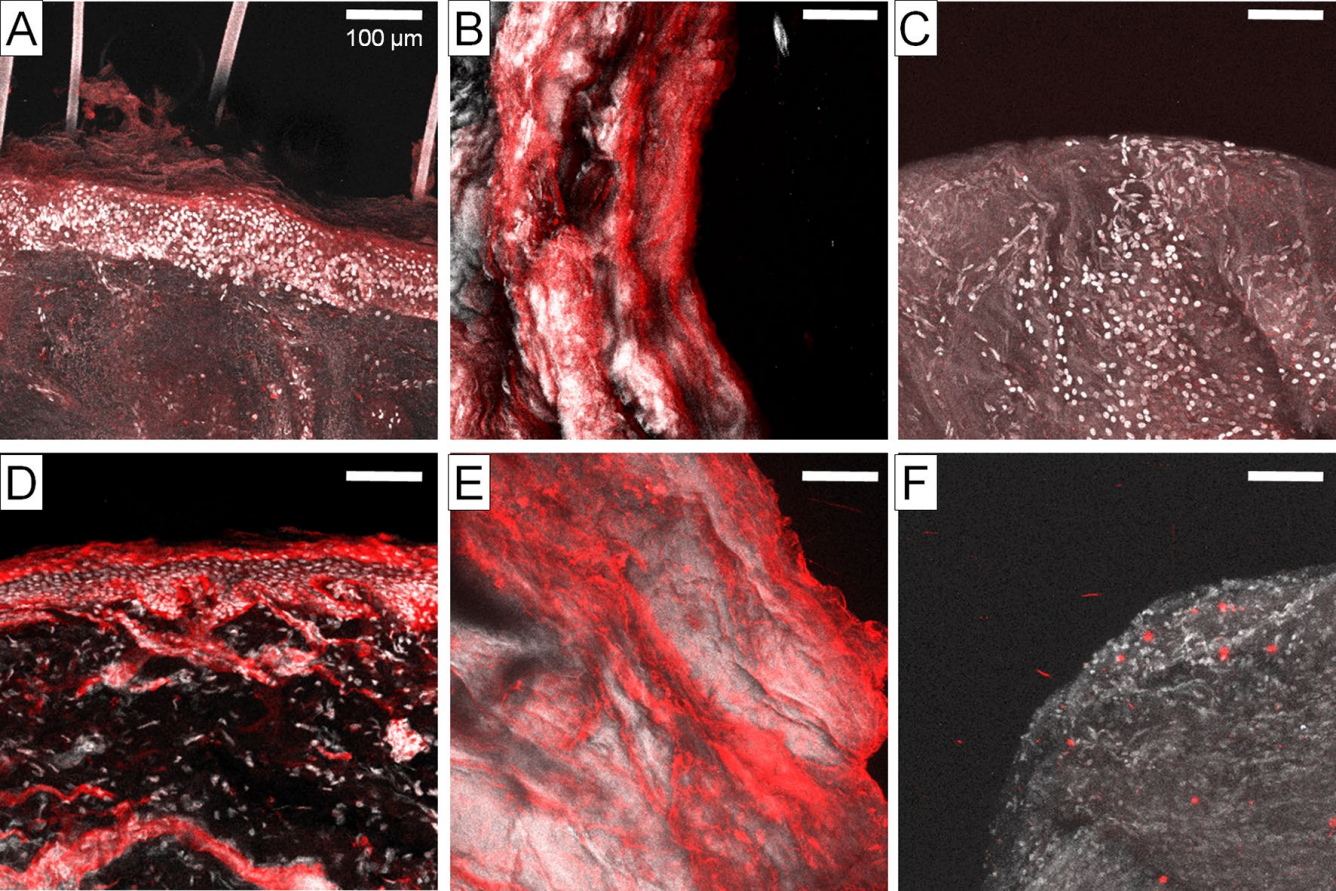
Cet-BPD PIC selectively localizes to human cholesteatoma and skin. (**A**) Cet-BPD PIC localizes to the epidermal region of fresh human skin, and (**B**) fresh human cholesteatoma tissue, but not (**C**) fresh middle ear mucosa tissue. (**D**) Cet-AF647 applied to epidermal region of fixed human skin, (**E**) fixed cholesteatoma tissue, and (**F**) fixed middle ear mucosa tissue confirms the pattern observed with Cet-BPD applied to fresh tissues.

When analyzing human skin, immune conjugate fluorescence localized to the epidermal layer while sparing the dermis (Fig. 3A). When analyzing cholesteatoma tissue, there was strong corollary localization within the surface tissues of the cholesteatoma sac (Fig. 3B). Figure 3C represents a control, using middle ear mucosa cultured with the PIC to demonstrate no localization or uptake in this tissue. Conventional fluorescent immunohistochemistry staining using Cet-AF647 in fixed tissues demonstrates the expected distribution for EGFR antibody affinity as a positive control for human skin (Fig. 3D), cholesteatoma sac (Fig. 3E) and middle ear mucosa (Fig. 3F). In all cases the distribution closely tracks with the localization patterns achieved with cholesteatoma explant tissues cultured with PIC in Fig. 3A-C.

## Discussion

This study is the first to demonstrate the potential for intraoperative imaging of cholesteatoma, and the first application in the head and neck region overall, using a novel application of an established imaging agent from the field of oncology. PIC concentrations of 100 µg/ml demonstrate ability to selectively localize epidermal tissues within the human middle ear, while safety tests in mice demonstrate no observed ototoxicity at a 10 × higher concentration. Taken together, the selective localization with in vitro cholesteatoma tissue culture and lack of ototoxicity are particularly valuable from a clinical applications perspective, as this opens the possibility of using this compound either intraoperatively or as a pre-treatment before surgery to help with intraoperative localization. Confirmatory tests with Cet-AF647 conjugate demonstrates localization reliability of this compound. While previous studies using fluorescent labeling for intraoperative tissue localization in the head and neck area have primarily focused on tissues with increased metabolic activity, primarily cancer cells, this study represents one of the first to successfully apply similar principles to benign disease without the aid of differential metabolic activity^24^. Furthermore, the unique photoactivation properties of PICs offers the potential for therapeutic targeted tumor ablation following appropriate localization^26–29^.

Limitations of the study include potential pitfalls associated with translation of current safety data from mice to humans, as well as difficulty replicating the conditions of cholesteatoma incubation in a clinical setting. While the homology of EGFR demonstrates 90% conservation across humans, dogs, mice and rats, key differences in subdomains I and III for the linear sequence of ErbB2 across species may affect validity of ototoxicity studies^37,38^. While it has been proposed in previous studies to maneuver around this limitation by using a “mouse Cetuximab” as the antibody base for evaluating off-target effects in the murine model, no such validated antibody currently exists^39^. Known side effects of Cetuximab relate primarily to various forms of skin rash, and across extensive investigations for a range of oncologic indications no concern for ototoxicity has yet been raised with this drug; alternative conjugates to humanized antibodies such a panitumumab may also be considered to reduce risk of other drug reactions^25,40–42^.

The optimal route of drug delivery for clinical application of this compound will also require further investigation. Given the 24-h time period required for metabolic uptake, localization and subsequent lyososomal cleavage of Cet-BPD, the compound would likely have to be administered a day in advance of any cholesteatoma surgery, thus requiring an additional clinic appointment^26,27^. More studies on systemic bioavailability would likely need to be conducted before IV administration could be considered feasible, and the option of an ototopical application may be more technically challenging and would require a provider experienced with otologic procedures to administer^25,43^.

The next steps in investigation should seek to evaluate the potential for selective photoactivation for cholesteatoma ablation, above and beyond the ability for selective imaging demonstrated in this study. These studies will likely require proof of concept in a murine xenograft model, in line with previous research supporting use of photodynamic therapy for localized, selective photoablation^26^. Further investigation of other PICs may also provide yet more effective routes of administration or more promising pharmacokinetics for this treatment avenue^22,23^. Finally, the current imaging system should be refined, since either a microscope- or endoscope-mounted system will eventually be necessary for clinical translation and use in the operating room.

## Supplementary Information

Supplementary Information 1.

Supplementary Information 2.

## Data Availability

The datasets used and/or analyzed during the current study available from the corresponding author on reasonable request.
