# Supplementary material for: A fluorescent photoimmunoconjugate for imaging of cholesteatoma
Source: Sci Rep. 2022 Nov 19;12:19905. doi: 10.1038/s41598-022-22072-9 (PMC9675863; doi:10.1038/s41598-022-22072-9)
Supplement: Supplementary file 1 — Supplementary Information 1. [file 41598_2022_22072_MOESM1_ESM.docx]

Supplementary Data: ABR and DPOAE raw data for murine hearing tests
